# Supporting Sustainability: A Philosophical Analysis of Certain Assumptions Underlying Occupational Therapy

**DOI:** 10.1177/00084174251317022

**Published:** 2025-02-17

**Authors:** Marie-Josée Drolet, Valérie Lafond

**Keywords:** Barriers, Obstacles, Occupational paradox, Paradigm, Sustainability, Barrières, durabilité, obstracles, paradigme, paradoxe occupationnel

## Abstract

**Background:** The relevance of supporting sustainability within occupational therapy is increasingly demonstrated and argued for. However, difficulties persist in supporting sustainability in practice, which presents occupational therapists with an occupational paradox. Why is sustainability still so difficult to include into occupational therapy practice? **Purpose:** The objective was to analyse certain assumptions underlying the profession that may constitute potential obstacles to the inclusion of sustainability in practice. **Method:** To identify and critically examine these assumptions, a philosophical analysis of five key concepts of the profession was carried out based on a triangulation of two philosophical methods. **Findings:** The results reveal that the five concepts analysed—person, occupation, environment, health, and justice—may act as barriers to support sustainability within the practice. **Conclusion:** A paradigm shift is therefore relevant, even necessary in the current climate emergency to mitigate its impacts. This is especially relevant as the climate crisis poses the greatest threat to health and endangers the ability of humans to engage in occupations essential for well-being and survival.

## Résumé

**Description.** La pertinence d’inclure la durabilité à la pratique de l’ergothérapie est de plus en plus démontrée et argumentée. Cependant, des difficultés persistent pour l’inclure dans la pratique, ce qui place les ergothérapeutes face à un paradoxe occupationnel. Pourquoi est-il encore si difficile d’inclure la durabilité à la pratique de l’ergothérapie? **But**. L’objectif était d’analyser certaines présuppositions au fondement de la profession qui constituent des obstacles potentiels à l’inclusion de la durabilité à la pratique. **Méthodologie.** Pour identifier et examiner de manière critique ces présuppositions, une analyse philosophique de cinq concepts clés de la profession a été réalisée sur la base d’une triangulation de deux méthodes philosophiques. **Résultats.** Les résultats révèlent que les cinq concepts analysés (personne, occupation, environnement, santé et justice) peuvent constituer des obstacles à l’inclusion de la durabilité à la pratique. **Conséquences.** Un changement paradigmatique semble dès lors pertinent, voire nécessaire, dans le contexte de l’urgence climatique actuelle, pour atténuer les impacts de cette crise. Cela est d’autant plus pertinent dans le contexte où la crise climatique est non seulement la plus grande menace à la santé, mais également une menace importante à l’engagement dans des occupations pouvant contribuer au bien-être, voire à la survie humaine.

## Introduction

This article is based on a June 2023 presentation by the first author at the Haute École de Travail Social et de la Santé in Lausanne, Switzerland, addressing the roles and challenges of occupational therapists amid the current climate crisis (Drolet, 2023). The presentation, refined through multiple discussions with the second author, was delivered again in May 2024 at the Canadian Association of Occupational Therapists’ annual conference in Halifax, Canada (Drolet, 2024). This article deepens the reflections behind such presentations and articulates a philosophical analysis on certain assumptions that may partly explain the difficulty many occupational therapists, at least in Canada, encounter in including sustainability into their practice.

This article is divided into five parts. First, we examine the main arguments justifying the inclusion of sustainability into occupational therapy practice. Second, we discuss what we have called the “occupational paradox” that occupational therapists may face when attempting to include sustainability into their professional practice, before synthesizing philosophical reflections that have been conducted within the profession in the context of climate change. Third, after identifying the question and objective of this article, we specify the methods we used to conduct the philosophical analysis underlying our ethical reflection. Fourth, we present the five findings that emerged from our analysis. Finally, we articulate a discussion on these results before concluding.

## Why Include Sustainability in Occupational Therapy?

Since Hooker's visionary call in 1972 to transform the occupational therapy training curriculum to better respond to the many challenges posed by the climate crisis and Wilcock's (1993) call to take a step back from our vision of occupational needs in this context, several authors and organizations have been inviting occupational therapists to include sustainability into their professional practice (e.g., Craik et al., 2024; Drolet & Turcotte, 2021; Ikiugu, 2008; Kiepek, 2024; Lafond & Drolet, 2021; Lieb, 2020; Persson & Erlandsson, 2014; Simó Algado, 2023; Smith et al., 2020; Ung et al., 2020; Wagman, 2014; Wilcock, 1993; World Federation of Occupational Therapists, 2018). In doing so, these authors invite occupational therapists to do mainly three kinds of actions: (a) support the reduction of ecological footprint of the healthcare system and their professional practice; (b) assist individuals and communities in their desire to engage in sustainable occupational transitions; and (c) support the resilience and adaptation of vulnerable individuals and communities directly confronted with climate hazards (Drolet & Turcotte, 2021; Taff et al., 2023). Therefore, occupational therapists may be led to participate in social struggles, advocate with various bodies, engage socially within an organization fighting against the climate crisis (Drolet & Lieb, accepted), and even take part in civil disobedience activities for the climate (Turcotte & Drolet, 2022). In addition, as noted by Ikiugu (2008), we recognize that sustainability is part of a broad set of interconnected global issues, such as diseases, poverty, social inequalities, global overpopulation, corruption, and wars.

There are several reasons for including sustainability in occupational therapy, and some of which are explained below. Firstly, health professionals, including occupational therapists, are concerned by the climate crisis because it poses the greatest threat to human health (World Health Organization, 2022). In occupational science or therapy, some authors argue that occupational therapists have a moral duty to respond to this crisis (Dennis et al., 2015; Drolet et al., 2020; Simó Algado, 2023). Rightly so, they think that occupational therapists must for instance minimize the healthcare systems’ ecological impact and help communities address the adverse consequences of the crisis.

Secondly, occupations contribute significantly to the climate crisis (Intergovernmental Panel on Climate Change [IPCC], 2022) and must therefore be integral to its resolution (Dennis et al., 2015; Turcotte & Drolet, 2020a). Occupational therapists are well positioned to assist individuals in adapting to the demands of the climate crisis (Dieterle, 2020) since they can be considered as experts in occupations and provide day-to-day support to individuals and communities in various occupational transitions.

Thirdly, the climate crisis significantly impacts human occupations worldwide through droughts, floods, and heatwaves, which disrupt occupational participation of an increasing number of human beings (Blakeney & Marshall, 2009; Pereira, 2009; Smith et al., 2020). Occupational therapists, with their expertise in enabling engagement in various occupations, could be key stakeholders in promoting health and well-being by supporting vulnerable individuals and communities in their resilience and adaptation to climate change (Taff et al., 2023; WFOT, 2022). For example, during climate hazards, occupational therapists could be part of field intervention teams to support individuals and communities in adequately responding to their occupational needs (e.g., access to nonflooded, air-conditioned places with adequate air quality, disaster response, relocation, etc.).

Finally, the climate crisis is linked to many injustices (McAdam & Rose, 2020; Williams, 2021), including intra or intergenerational occupational injustices (Drolet et al., 2020; Símo Algado & Townsend, 2015), since those who suffer the most from the climate crisis are often not its main contributors. Considering that occupational justice is valued in occupational therapy (Désormeaux-Moreau & Drolet, 2019; Drolet, 2014; Drolet & Désormeaux-Moreau, 2019) and that occupational therapists can work towards occupational justice, such injustices should concern them.

### An Occupational Paradox

The climate crisis presents occupational therapists with what we have called an “occupational paradox.” Considering that humans need the environment to carry out their occupations, and that human occupations paradoxically destroy the environment, the occupational paradox is as follows: the occupations carried out by humans destroy the condition of possibility of their occupations. Occupational therapists, who traditionally view occupations positively, realize the profound impact that human occupations have on ecosystems amid the escalating climate crisis. Our health, well-being and survival hinge on the natural environment, which is paradoxically degraded and destroyed by the very occupations that depend on it for their existence. In fact, the climate crisis is linked to an unprecedented ecocide (Cabanes, 2017) caused by human occupations (IPCC, 2022). This is especially true for occupations carried out in capitalist and neoliberal societies (Abraham, 2019; Parrique, 2022), which tend to operate under the erroneous assumption that natural resources are infinite, thus blatantly ignoring the reality of our finite world. However, occupational engagement necessitates an environment, as no occupation can occur without it.

Occupational therapists, with their expertise in the dynamics between individuals, occupations, and environments (Law et al., 1996), can effectively address this occupational paradox (Turcotte & Drolet, 2020b). How exactly? Notably, by including sustainability in their practice, as suggested by several authors and professional organizations (e.g., Craik et al., 2024; Drolet & Turcotte, 2021; Ikiugu, 2008; Kiepek, 2024; Lafond & Drolet, 2021; Lieb, 2020; Persson & Erlandsson, 2014; Simó Algado, 2023; Smith et al., 2020; Ung et al., 2020; Wagman, 2014; WFOT, 2018; Wilcock, 1993). In addition, they can participate in the deconstruction of various systems of oppression that limit nowadays the occupational participation of so many individuals and communities, and cause various occupational injustices (Hammell, 2020), which are exacerbated in the context of the climate crisis (INSPQ, 2023).

Although few empirical studies have so far documented the sustainable practices carried out by occupational therapists (Fawkes et al., 2024; Lafond & Drolet, 2021), the literature suggests various means to support sustainability in occupational therapy. Some authors believe, for example, that occupational therapists can help decrease the ecological footprint of their practice, or of the health system (e.g., Lafond & Drolet, 2021; Ordway et al., 2018). Others believe that occupational therapists can support individuals and communities to attain sustainability (e.g., Dennis et al., 2015; Dieterle, 2020; Drolet & Lafond, 2022; Hocking & Kroksmark, 2013; Thiébaut et al., 2023). Finally, some believe that occupational therapists can support individuals and communities to adapt to the climate crisis (e.g., Pereira, 2009; Rushford & Thomas, 2016), while others prioritize addressing the root causes of poverty, food insecurity, and injustice (e.g., Hellwig, 2023; Símo Algado & Townsend, 2015).

### Philosophy, Occupational Therapy, and Sustainability

In the context of contemporary reflections on sustainability in occupational therapy, some authors have developed reflections of a philosophical nature, more precisely ethical reflections on various subjects. For example, Persson and Erlandsson (2002, 2014) carried out an ethical reflection on the industrial society that led to the creation of the concept of ecopation, which refers to occupations carried out with an ecological concern. For their part, Drolet et al. (2020) conducted an ethical reflection that led to the conceptual clarification of five occupational concepts, namely needs, desires, choices, rights, and duties, as well as the creation of the concept of intergenerational occupational justice. Ung et al. (2020) realized an ethical reflection that led to the concept of eco-occupation to better support intergenerational occupational justice. Turcotte and Drolet (2022) proposed a tool to aid ethical reflection to support occupational therapists wishing to engage in civil disobedience activities in the context of the fight against the climate crisis. For their part, Drolet and Lafond (2022) conducted an ethical reflection on the legitimacy (or not) of advocating for sustainability and intergenerational occupational justice at the expense of the client-centered approach in a clinical context. That said, to date, no philosophical analysis has examined the possible conceptual barriers to the inclusion of sustainability in occupational therapy, taking care to examine their epistemology and axiology.

### Research Question and Objective

Despite the rich diversity of perspectives and the ethical and scientific justifications for integrating sustainability into practice, many Canadian occupational therapists still find it challenging to do so (Chan et al., 2020; Désormeaux-Moreau et al., 2021; Du et al., 2024; Lafond & Drolet, 2023), particularly in clinical and educational contexts. Why is that?

In 1997, do Rozario asserted the need to transform the paradigms of the profession to include sustainability into occupational therapy practice. Following this idea of paradigm shift, as stated earlier, the concepts of ecopation (Persson & Erlandsson, 2014) and eco-occupation (Ung et al., 2020) have been proposed. In response to the climate crisis, distinctions have been made between occupational needs, desires, choices, rights, and duties, alongside the proposition of the concept of intergenerational occupational justice to capture occupational injustices between generations (Drolet et al., 2020). These new concepts seem to indicate that there is a lack of solid conceptual support within the profession to better support sustainability in occupational therapy.

Furthermore, other authors have identified additional barriers that explain the difficulty of including sustainability in occupational therapy. Lafond and Drolet (2023) argue that personal, professional, organizational, and societal barriers may influence the adoption of sustainable practices by occupational therapists within their practice. For instance, living far from the workplace, adopting a client-centered practice while respecting unsustainable lifestyles (Drolet & Lafond, 2022), complying with unsustainable organizational policies, and the lack of consideration for sustainability in care settings have been identified as barriers to a sustainable practice of occupational therapy (Lafond & Drolet, 2023). Also, Chan et al. (2020) assert that barriers to adopting sustainable lifestyles exist at both individual and institutional/systemic levels, including physical and socioeconomic challenges, as well as the low prioritization of sustainability in the healthcare sector's political agenda. Désormeaux-Moreau et al. (2021) assert that promoting and supporting sustainable occupational transitions could threaten collective professional identity. Du et al. (2024) assert that barriers to sustainability exist at both individual and systemic levels, and include a lack of practitioner guidelines for promoting sustainability within their specific mandates and a capitalist and colonialist system that perpetuates injustices. Although the WFOT (2018) has developed a guide to include sustainability in occupational therapy practice, it can indeed be challenging for many occupational therapists to connect these general guidelines with their daily practice, considering that their mandate is generally not related to sustainability. Moreover, considering that the capitalist economy values perpetual GDP growth in the context of finite natural resources, many economists believe with reason that this vision of production is not sustainable (Abraham, 2019; Parrique, 2022) and perpetuates colonial injustices (Williams, 2021).

Still, given the urgency to act and the extent of the current and future occupational consequences and injustices, this inertia is both surprising and worrying. Therefore, the objective of this article is to philosophically analyse fundamental paradigmatic assumptions in the occupational therapy profession that may hinder the inclusion of sustainability by examining the epistemological and axiological dimensions of different key concepts of the profession. In addition to the barriers to including sustainability in occupational therapy described earlier, following do Rozario (1997) who suggested that a paradigm shift was required to do so, the intuition behind this article is that there may be conceptual barriers within our theories, models, and conceptual frameworks that also partly limit the inclusion of sustainability into occupational therapy practice.

## Method

As listed in Table 1, to carry out our philosophical analysis, five steps were undertaken. Although these steps are listed in Table 1 in a linear sequence, they have been in fact carried out iteratively, as the following paragraphs specify.

**Table 1:** The Followed Iterative Steps to Conduct the Philosophical Analysis

1. Choosing the relevant corpus of texts to analyse in today's Canadian context
2. Selecting the key concepts to be philosophically analysed and their assumptions
3. Analysing the key concepts using philosophical methods and summarizing the analysis
4. Comparing this summary with a sustainable vision of the profession for each key concept
5. Synthesizing the conclusions of the comparison for each of the analysed concepts

Firstly, we have chosen central texts that are relevant to the current practice of the profession in Canada, both in clinical practice and in teaching. The aim at this step was not to be exhaustive in the selection of texts corpus, but to choose texts that are, to our knowledge and experience, central and foundational to the contemporary profession's usual vision in Canada. As specified below, this approach is typical of the hermeneutic method used in philosophy (Gadamer, 2001; Husserl, 1970).

Secondly, we selected key concepts of the profession that could possibly hinder the inclusion of sustainability in occupational therapy practice. More specifically, we imagined occupational therapy as it could have been if, as a profession, we had accepted Hooker's invitation in 1972 to develop preventive occupational therapy, that is, occupational therapy that could prevent the deterioration of ecosystems by supporting the engagement of individuals, communities, companies, and governments in sustainable occupational transitions. From this vision of the profession, we asked ourselves which of the foundational and key concepts of the profession could constitute barriers to such a project. Five key concepts emerge from the principles of sustainable professional practice: person, environment, occupation, health, and justice. Considering that these key concepts are fundamental to occupational therapy in any practice context, this choice seemed especially relevant.

Thirdly, we conducted a philosophical analysis of these key concepts and their foundational paradigmatic assumptions. Philosophy, as a discipline that creates concepts (Deleuze & Guattari, 1991) and clarifies them through the use of intellectual faculties (Wittgenstein, 2003), has developed methods for conducting conceptual analyses. Philosophers primarily use two methods: the analytical method (e.g., Russell, 2009; Wittgenstein, 2003) and the hermeneutic method (e.g., Gadamer, 2001; Husserl, 1970). While the first focuses on the logical clarity and argumentative structure of a text, ensuring coherence of its concepts, the second emphasizes the context and period in which the concepts are studied. More specifically, to carry out a philosophical analysis using the analytical method, the analyst must firstly “proceeds backwards from a body of knowledge to its premisses, and, secondly, […] proceeds forward from the premisses to a reconstruction of the original body of knowledge” (Hager, 2003, p. 310). In other words, the analytical method requires the analyst to deconstruct the body of knowledge to identify the conceptual assumptions of interest (Step 2), then to synthesize them (Step 4) in order to compare them (Step 5) with a sustainable vision of the profession as proposed by Hooker (1972). Moreover, to carry out a philosophical analysis using the hermeneutic method, the analyst must consider a specific cultural context and a specific period in order to identify the corpus of texts of interest (Step 1). In our case, the aim was to identify the assumptions of current occupational therapy practice in a Canadian context (Step 1).

During our analysis, a reflexive balance was used to triangulate these two common philosophical methods (Savoie-Zajc, 2009) with a greater emphasis on the analytical method. In other words, from the ideal of sustainable occupational therapy practice, we conducted a philosophical analysis of these five key concepts using these established philosophical methods. More specifically, we analysed these concepts from a philosophical perspective, focusing on their epistemological (learning-based perspective) and axiological (values-based perspective) foundations and underlying conceptual assumptions, using the analytical tools that philosophy provides.

Fourthly, to compare the summary of our philosophical analysis with a sustainable vision of the profession, the following actions were carried out: (a) after identifying the concepts under study and their conceptual assumptions, we analysed them one by one from the vision of preventive occupational therapy, as suggested by Hooker (1972), that is, sustainable occupational therapy practice, to assess whether the contemporary conception of each of them, in the Canadian context, supports sustainability; (b) if this was not the case, we asked ourselves how these concepts should be conceived to better support sustainable occupational therapy practice, thus outlining the gap between our contemporary Canadian vision of these concepts and the one that should be promoted to better support such professional practice.

Fifthly, we synthesized the conclusions of the comparison for each of the analysed concepts. Therefore, analysing concepts from a philosophical perspective involves dissecting them to identify inconsistencies between current professional practices and sustainable ones, thus highlighting both epistemological and axiological gaps between them. This also implies considering the contemporary context in which the profession is practiced, comparing the ideal of sustainable professional practice with current practice, at least in Canada and to the best of our knowledge.

## Results: A Journey Leading to Five Discoveries

Five findings emerged from our philosophical analysis. Precisely, to better include sustainability in their professional practice, occupational therapists should move from: (a) an individual approach toward an intergenerational and interspecies population-based approach; (b) a positive view of occupations toward a more inclusive, nuanced, even negative view of occupations; (c) an instrumental and anthropocentric view of the environment toward an intrinsic and ecocentric view of the environment; (d) a reductive view of health to a broader and ecosystemic view of health; and (e) an individualistic vision of justice centered on today's human beings to a collective vision of justice centered on today's and tomorrow's living beings. In other words, the philosophical analysis of the five concepts led us to these five findings: one finding per analysed concept.

The results presented in this section are based on the philosophical analysis of central texts usually used, to our knowledge and experience, in both occupational therapy clinical practice and teaching in Canada (see Table 2). Although by no means exhaustive, these texts have been chosen because they are central and largely cover the contemporary vision of the occupational therapy profession in Canada.

**Table 2:** The Analysed Concepts and the Corpus of Texts Studied

Analysed concepts (*n* = 5)	Studied texts’ corpus (*n* = 14)
Person, occupation, environment, health, justice	ACOTRO, ACOTUP & CAOT, 2021; Christiansen et al., 2015; Cloutier et al., 2018; Durocher, 2017; Egan & Restall, 2022; Hammell, 2017; Iwama, 2006; Kielhofner, 2009; Laliberte Rudman et al., 2022; Law et al., 1995; Taylor et al., 2024; Townsend & Polatajko, 2013; Wilcock, 1998; Wilcock & Hocking, 2015

### Concept 1: The Person. Moving From an Individual Approach Toward an Intergenerational and Interspecies Population-Based Approach

Even though the new Canadian Model of Occupational Participation (Egan & Restall, 2022) focuses on collaborative relationships with individuals, families, groups, communities, and populations, the approach underlying day-to-day practices of many Canadian occupational therapists remains predominantly person-centered (e.g., Christiansen et al., 2015; Kielhofner, 2009; Law et al., 1995). As such, occupational therapists generally focus on a person and sometimes their loved ones, their values, interests, needs, and occupations. Even when occupational therapists adopt a population-based approach, they typically focus on the needs, interests, and rights of human beings, rather than those of nonhuman animals or ecosystems (ACOTRO, ACOTUP & CAOT, 2021). However, including sustainability in occupational therapy practice may require adopting a population-based approach that is both intergenerational (Drolet et al., 2020; Drolet & Lafond, 2022; Thiébaut et al., 2020) and interspecies (Bilat, 2023; Kiepek, 2024), a concept which escapes many occupational therapists currently.

Moreover, in Western societies, the prevailing concept for a person is that of the *homo economicus*, according to which so-called rational individuals are to maximize their satisfaction and wealth by using resources efficiently (Hammell, 2020). In fact, occupational therapists frequently aim to enhance people's participation in occupations and improve their overall occupational performance, productivity, and integration into societal and economic structures (Persson & Erlandsson, 2002). Much like other professionals in Western societies, Canadian occupational therapists operate within a productivity-driven and growth-focused framework (Abraham, 2019; Parrique, 2022), which often fails to challenge neoliberal capitalism (Taff & Putnam, 2022).

Therefore, many occupational therapists find it challenging to prioritize occupations beyond productivity (Hammell, 2020), to avoid becoming a mere bureaucrat within the state's apparatus (Drolet et al., 2020), or even a slave to capitalist ideologies and organizations (Turcotte & Holmes, 2021). This individualistic, capitalist, and neoliberal vision of personhood indeed permeates occupational therapy (Clouston, 2014; Hammell, 2020; Newman & Lawler, 2009; Taff & Putnam, 2022) as well as the healthcare system (Drolet, Lalancette, & Caty, 2020; Leicht et al., 2009). So, practitioners are not encouraged to adopt a long-term perspective that considers not only the needs, interests, and rights of present and future humans, but also those of other living beings and ecosystems, even though it is crucial to do so.

In summary, a philosophical analysis of the concept of person shows that understanding this concept from the perspective of sustainability requires a certain epistemological shift (a new and different understanding of this concept which, e.g., considers future humans) and axiological shift (valuing not only humans, but also nonhuman animals and ecosystems).

### Concept 2: Occupation. Moving From a Positive View of Occupations Toward a More Inclusive, Nuanced, or Even Negative View of Occupations

Occupational therapy is based on a view of humans as inherently occupational beings, engaging in occupations that are essential for survival, promote health, fill time, facilitate social connections, or add meaning to life (e.g., Christiansen et al., 2015; Drolet, 2014; Kielhofner, 2009; Taylor et al., 2024; Townsend & Polatajko, 2013). While the negative aspects of some occupations have been explored (Kiepek et al., 2019; Twinley, 2021), it is widely accepted that occupations generally enhance health and well-being (Laliberte Rudman et al., 2022; Townsend & Polatajko, 2013; Wilcock & Hocking, 2015). Overall, the profession maintains a positive view of occupations because they are often linked to health, well-being, and human fulfillment.

Moreover, occupational therapists are primarily tasked with supporting both occupational participation and performance. Many occupational therapists find it difficult to work with people who are reluctant to engage in self-care and household tasks or participate in productive activities such as studying and working. Indeed, many occupational therapists view the world, and human beings, through an occupational or productivist bias (Drolet & Maclure, 2016). Considering that degrowth within the occupational therapy profession is not automatically accepted (Turcotte & Drolet, 2020a), evident in healthcare (Ouimet et al., 2020), or apparent in the broader social context (Abraham, 2019; Parrique, 2022), supporting occupational degrowth is therefore far from a given within the profession.

However, including sustainability into practice presents a new perspective on occupations, one that is both more negative and more inclusive. Considering that occupations are the cause of the climate crisis (IPCC, 2022), occupational therapists are increasingly aware of their detrimental impacts on the health of all living beings and on ecosystems. In fact, occupations within capitalist and productivist societies, which prioritize economic growth above all else (Abraham, 2019; Parrique, 2022), are the main contributors to ecocide, that is, the irreversible destruction of ecosystems, including both renewable and nonrenewable natural resources (Cabanes, 2017). This illustrates the occupational paradox many occupational therapists encounter when attempting to include sustainability into their practice. As a reminder, the occupational paradox is as follows: the occupations carried out by humans destroy the condition of possibility of their occupations. Should therefore the occupational therapist support engagement in occupations that contribute to destroying the environment, knowing that in doing so, he or she contributes to limiting the occupational engagement of other present or future humans as well as an increasing number of nonhuman animals? To what extent is it ethical to support the occupational engagement of certain individuals when it impacts that of other humans and nonhuman animals? What are the occupational duties of humans toward present and future generations of humans and nonhuman animals? These ethical questioning constitute an ethical dilemma that may be faced by some occupational therapists (Drolet & Lafond, 2022).

Moreover, including sustainability into practice can help occupational therapists realize that nonhuman animals are also occupational beings, since they engage in occupations. This means that the occupational nature is not unique to humans (Kiepek, 2024). Indeed, nonhuman animals perform a variety of daily activities essential for survival and other species-specific purposes. Recognizing that both humans and nonhuman animals engage in occupations expands the scope of occupational therapy to include interspecies considerations and address diverse occupational needs (Bilat, 2023; Kiepek, 2024).

In summary, a philosophical analysis of the concept of occupation shows that understanding this concept from the perspective of sustainability requires a certain epistemological shift (a new and different understanding of this concept which, for example, includes nonhuman animals as occupational beings as well as a more negative view of occupations considering their negative impacts on the environment) and axiological shift (valuing certain occupations, i.e., those that regenerate ecosystem, at the expense of occupations that destroy it).

### Concept 3: The Environment. Moving From an Instrumental and Anthropocentric View of the Environment Toward an Intrinsic and Ecocentric View of the Environment

To our knowledge, except for the Kawa Model (Iwama, 2006), the concept of the environment in occupational therapy primarily holds value as it enables human occupation (e.g., Christiansen et al., 2015; Cloutier et al., 2018; Kielhofner, 2009; Law et al., 1995; Townsend & Polatajko, 2013; Wilcock & Hocking, 2015). Indeed, it has instrumental value, for it is a means to human ends. In this regard, occupational therapy's view of the environment is both instrumental and anthropocentric. Such vision of the environment is obviously not unique to occupational therapy profession. It permeates Western culture as a whole, at least since the Greek philosophers of Antiquity. Indeed, this view has its origins in Western dualism, particularly in the philosophies of Plato and the 17th-century thinker Descartes (Plumwood, 2002). This dualistic conception posits that nature is opposed to and subordinated to culture, similar to how the body is viewed in relation to the mind and how women are distinguished from men. In each case, the former is considered inferior to the latter (Plumwood, 2002; Warren, 2009). Christianity conveys that God entrusted the environment to humanity for both enjoyment and exploitation to meet human needs.

However, including sustainability in occupational therapy requires a completely different vision of the environment. Several sustainability thinkers within the profession rightly criticize this instrumental and anthropocentric vision of the environment, advocating instead for an intrinsic and ecocentric view, which posits that the environment has intrinsic value beyond its utility (Domenjoud & Clavreul, 2023; Thiébault, 2018; Thiébault et al., 2020). Consequently, the environment, recognized as a rights-bearing entity, warrants respect and ethical consideration, extending our responsibilities beyond just human beings (Jonas, 1979). Moreover, adopting an ecocentric view of the environment expands our ethical considerations to include all living beings, thus recognizing the interdependence and interconnectedness within the natural world (Shiva, 2016). In doing so, occupational therapists are invited to explore Indigenous, African, and Asian perspectives on the environment. Such perspectives have long recognized the inherent interdependence and interconnectedness of life, which are vital for supporting health and well-being (Basile, 2018; Du et al. 2024; Kiepek, 2024; Lieb, 2022; Plumwood, 2002; Shiva, 2016; Suzack, 2015).

In summary, a philosophical analysis of the concept of the environment shows that understanding this concept from the perspective of sustainability requires an important epistemological shift (it requires moving away from Western epistemologies, which generally have an instrumental and anthropocentric view of the environment, to embrace an ecocentric view of the environment) and axiological shift (this requires giving intrinsic value to the environment, thus drawing from Indigenous, African, and Asian ethics).

### Concept 4: Health. Move From a Reductive View of Health to a Broader and Ecosystemic View of Health

The analysis of our text corpus (e.g., ACOTRO, ACOTUP & CAOT, 2021; Christiansen et al., 2015; Law et al., 1995; Townsend & Polatajko, 2013; Wilcock & Hocking, 2015) reveals an individualistic vision of health. As noted by Gerlach et al. (2018) and Lieb (2020), the individualistic vision of Western society is also reflected in contemporary occupational therapy services. Indeed, individualism “involves viewing various social issues and phenomena, including occupation, as primarily residing in and being shaped through individuals” (Gerlach et al. 2018, p. 36). Viewed this way, healthcare becomes an individual experience through the system. In fact, not only is the healthcare system individualistic, but it also perpetuates a reductive perspective on health (Parent & Jouquan, 2013; Rocca & Anjum, 2020). In this reductive view, illness is solely considered as a physical or biological disease, with its effects analysed by examining individual body parts (Rocca & Anjum, 2020). Indeed, health care is often compartmentalized into distinct sectors, such as physical and mental health, which impedes a holistic and comprehensive approach to health. This perspective on health derives from Descartes’ dualistic conception, which separates the body and mind, and from a mechanistic approach that treats body parts independently of their context (Rocca & Anjum, 2020). The prevailing view of health inadequately addresses illness because it overlooks the holistic aspects of an individual, neglecting to consider their habits, social and psychological factors, and environmental context (Rocca & Anjum, 2020). Such view therefore overlooks the structural and social determinants of health (Gerlach et al., 2018), as well as environmental determinants. Although occupational therapists generally value holism (Drolet & Désormeaux-Moreau, 2019; Finlay, 2001), given the practice contexts that prioritize speed and performance (Drolet, Lalancette, & Caty et al., 2020), it remains challenging for many of them to uphold this value on a daily basis (Drolet & Désormeaux-Moreau, 2019).

However, including sustainability in occupational therapy practice requires shifting from a narrow, individual-focused view of health to a broader vision. To this end, the One Health approach is an interesting way of rethinking the perspective on health (One Health High-Level Expert Panel [OHHLEP], 2022). One Health “is an integrated, unifying approach that aims to sustainably balance and optimize the health of people, animals, and ecosystems. It recognizes the health of humans, domestic and wild animals, plants and the wider environment are closely linked and inter-dependent” (OHHLEP, 2022, p. 11). In other words, this approach recognizes that both humans and nonhuman animals require a healthy and safe environment to thrive. Indeed, humans and nonhuman animals alike breathe air, drink water, and eat crops from the land, which emphasizes the interdependence with the environment. If such elements are negatively impacted by pollution, or climate change, so is health. Then, including sustainability within the profession involves recognizing the interdependence of humans and nonhuman animals with their environments. It requires reflecting on how occupations can support and adversely affect health, thereby addressing the occupational paradox. Indeed, humans and nonhuman animals are part of nature, and the natural environment supports them as well as exists within them. It follows that if the natural environmental is unhealthy, human and nonhuman animals will be unhealthy.

In summary, a philosophical analysis of the concept of health shows that understanding this concept from the perspective of sustainability requires a major epistemological shift (a decentered conception of the health of human individuals and populations to include the health of nonhuman animals and ecosystems, considering the interdependence between the health of all living beings) and axiological shift (valuing the health of all living beings not just that of humans).

### Concept 5: Justice. Move from an Individualistic Vision of Justice Centered on Today's Human Beings to a Collective Vision of Justice Centered on Today's and Tomorrow's Living Beings

Since its introduction by Wilcock (1998), the concept of occupational justice has become a significant ethical principle in occupational therapy and occupational science (e.g., Durocher, 2017; Hammell, 2017). Generally, in occupational therapy and occupation science, justice is conceptualized in terms of individual occupational rights, rather than collective rights. Additionally, what generally interest authors are the occupational rights of today's human beings, not those of future generations. Furthermore, occupational rights are considered human rights, exclusive to humans and not applicable to nonhuman animals. In sum, the profession's concept of justice is individualistic and specist (Singer, 2017), focusing only on contemporary human concerns.

However, to include sustainability into occupational therapy, the practice must expand its vision of justice to be collective, antispecist, and ecocentric, moving away from an individualistic, specist, and anthropocentric approach. In other words, it requires the recognition of collective rights that encompass all current and future living beings. Thus, adopting a perspective that is both collective and encompasses intergenerational and interspecies considerations is essential. This is what intergenerational occupational justice offers (Drolet et al., 2020), which is also interspecies justice (Bilat, 2023). Indeed, intergenerational occupational justice considers the relationships between current and future human generations, while also addressing the rights of nonhuman animals and the environment. It invites current generations of human beings to engage in eco-responsible occupations (occupational duties) to respect the occupational rights of present and future generations of human (Drolet et al., 2020) and nonhuman animals (Bilat, 2023). Thus, it requires not only considering the occupational rights of individuals but also the occupational duties that humans have toward present and future generations of all living beings (Bilat, 2023; Drolet et al., 2020).

In summary, a philosophical analysis of the concept of justice shows that understanding this concept from the perspective of sustainability requires a major epistemological shift (a new vision of occupational justice that is collective, antispecist, and ecocentric) and axiological shift (expanding the circle of our ethical considerations to include both human and nonhuman animals of today and tomorrow, as well as ecosystems).

## Discussion

The objective of this article was to philosophically analyse fundamental assumptions in occupational therapy that might hinder the inclusion of sustainability into the profession. Thus, five key concepts were identified and analysed. The five findings outlined above indicate that foundational assumptions within occupational therapy may indeed create conceptual obstacles to including sustainability into practice. In summary, it emerges from the analysis that a paradigmatic change is required, including epistemological (the way we define concepts and the theories on which these definitions are based) and axiological (the importance we give to various elements, including values such as the human person, the natural environment, occupational engagement, health, and justice) changes. In order words, do Rozario (1997) was right: a paradigm shift seems to be required to achieve the inclusion of sustainability into occupational therapy practice.

Of course, there are probably other reasons for the difficulty of including sustainability in practice, as reported in the introduction, which we will explore below. But first do our findings echo similar reflections published to date? To the best of our knowledge, the reflection proposed here is both unique and pioneering. Still, the conclusions drawn here align with observations from previous research, as evidenced by the references in the preceding section.

That said, the context in which the profession operates, as discussed in this article, may also account for the difficulty of including sustainability in occupational therapy. Generally, healthcare and education systems, as well as Western societies, continue to adopt sustainability practices slowly (Du et al., 2024). This mainly stems from the alignment of these systems with the profession's core assumptions about human beings, occupation, environment, health, and justice. More specifically, Western societies, such as Canada, predominantly uphold individualistic and productivist values, as opposed to collectivist and degrowth-oriented approaches. These values tend to be neither proactive nor effective in protecting and preserving planetary ecosystems. As a result, they struggle to shift from a reductive vision of health to an interactionist and holistic vision, such as the one proposed by the One Health approach (OHHLEP, 2022). They still place little value on health promotion and disease and accident prevention, which are nonetheless proven to be effective. For example, although evidence strongly supports the benefits of addressing social determinants of health, public resources allocated to prevention and promotion remain insufficient. We know that egalitarian societies are healthier, happier, and have less crime (Wilkinson & Pickett, 2010), yet our societies are far from being egalitarian. Furthermore, Western societies contribute significantly to various global injustices, including occupational injustices, by basing their wealth production on the exploitation and dispossession of the less fortunate (Harvey, 2005; Parrique, 2022). A profound global injustice underpins the wealth of Western societies: not only do they exploit the poorer and middle-income countries to accumulate wealth (Parrique, 2022), but they also export their waste to these countries, exposing the inhabitants to health risks—a practice commonly known as toxic colonialism (Atapattu et al., 2021; Kiepek, 2024; Williams, 2021). Moreover, Western societies exacerbate climate injustices; those who emit the most greenhouse gases and significantly contribute to the climate crisis are often the least affected by it (Jamieson, 1992; Roser & Seidel, 2017; Shue, 2014; Williams, 2021).

Although occupational therapists, like other healthcare professionals and indeed all humans, are ethically obliged to fight the climate crisis (Drolet et al., 2020), some foundational assumptions of the profession may hinder this fight, as examined here. That said, sustainability has the potential to positively evolve the profession, enabling occupational therapists to contribute to a fairer world in terms of occupation, for both present and future generations of living beings. Sustainability also provides occupational therapists a vital role in safeguarding the present and future of humanity and numerous other species. Thus, we hope that the invitation extended by do Rozario in 1997 to engage in a paradigm shift will be heeded. We also hope that the challenge posed to occupational therapists by Hooker in 1972 to transform our curricula will be addressed, as well as Wilcock's 1993 call for humanity to engage in a serious and reasoned reevaluation of our occupational needs. It is indeed important to differentiate between genuine occupational needs of human beings and the numerous desires portrayed by the industry as necessities, which are often misleading (Drolet et al., 2020). To meet the occupational needs of present and future generations, both human and nonhuman animals, we must collectively restrain our immediate desires; we're talking here to polluting companies, governements and the world's wealthy citizens, who hyper-consume and are responsible for the crisis. Overindulging these occupational desires harms the environment, which is an essential condition for all occupational occupations. Recent significant literature on sustainability in occupational therapy suggests a potential transformation of both professional practices and the profession itself. Like a chameleon, are we transforming the profession to better adapt to the climate crisis and its numerous occupational challenges? Isn’t it our collective duty to rethink and reimagine tomorrow's profession today, to better prepare present and future generations for these major challenges?

## Strengths and Limitations

This philosophical analysis presents strengths and limitations. In terms of strengths, this paper is, to our knowledge, the first to offer a philosophical analysis of certain central assumptions of the profession that can act as a barrier to support sustainability in occupational therapy practice. Indeed, the concepts of person, environment, occupation, health and justice, which are key concepts of the profession, have been analysed, making it possible to clarify the occupational paradox facing occupational therapists, and to present possible solutions to counter it. As for limitations, since this article is based on the authors’ reflections following Hooker's invitation and vision (Hooker, 1972), other assumptions that may support sustainability in occupational therapy might have been overlooked due to ethical blindness. This article is therefore intended as a starting point for examining how the profession's key concepts and values are perceived and conveyed, and how they impede sustainability within the profession.

## Conclusion

This article has identified some of occupational therapy's assumptions that present themselves as conceptual obstacles to the inclusion of sustainability in the profession. A paradigm shift is therefore relevant, even necessary, in the context of the current climate crisis, because not only is it the greatest threat to health, but also a major threat to the very possibility of humans engaging in occupations that contribute to their well-being and survival. This article has highlighted the importance of adopting a philosophical perspective to question key concepts of the profession. By analysing these concepts form a philosophical stand, we can evaluate whether they align with the vision necessary to address the climate crisis. This provides a perspective to prevent conceptual barriers to including sustainability in occupational therapy, thereby possibly reducing occupational and climate injustices. Since human health is intrinsically linked to planetary health (OHHLEP, 2022), it is therefore important to counter such injustices to ensure a healthy environment that supports occupational engagement for present and future generations (Drolet et al., 2020) of all living beings.

## Key Messages

- Many Canadian occupational therapists struggle to incorporate sustainability into their practice.
- Certain presuppositions at the foundation of the profession present themselves as obstacles to the inclusion of sustainability in occupational therapy.
- A paradigmatic revolution is needed to bring sustainability into occupational therapy practice.
